# Failing Eyesight

**Published:** 1861-10

**Authors:** 


### FAILING EYESIGHT.

"When ought I to begin to use spectacles?" is the inquiry of all who, having passed the up-hill of life, are making their way downward on the other side. The necessity of glasses comes sooner to some than others, according to the variety of circumstances and conditions which are allotted to human kind; hence it would be unwise to name any particular age. The sad necessity, however, comes with timely warnings, each successive one becoming more and more decisive. To the hearty, healthy, temperate and strong, the "symptoms" of needed spectacles begin to make their unwelcome appearance about the age of fifty years. To our wives, so unwisely industrious as to stitch, stitch, stitch, until the bell strikes midnight, under the unanswerable plea, "I have to do it," the indications of failing eyesight are ten years earlier; but whether at fifty or forty, they are the same. Among the very first is an instinctive preference for the larger print; next, and before we are aware of it, it is found that a habit has been formed of selecting the lightest spot in the room for reading or fine sewing; after a while, a year or more, there is either a disposition to put the newspaper farther from the eye, or there is some little adjustment of it necessary in order to enable one to read with entire comfort; after a while, there is a disposition to stop reading for a second or two, and wink the eyes several times, or to rest them by looking at something at a distance, as if to gain more strength to see distinctly the lines and letters read; then comes the feeling of aid given to the eye by placing the finger near the line read, as if to steady the paper, or as if to enable the eye to get at the line more readily. Reader, when you find yourself reading by the aid of your finger, thus, you are beginning to be an old man; "gray hairs are upon you;" your sight has begun to fail you, and you should at once purchase glasses. Those made of Brazilian pebble, being natural glass, are the best, because they are not so easily broken, are not readily scratched, and do not gather moisture so soon, hence do not need to be so often wiped; they are more expensive than the common kind. Common glasses, in blue steel frames, cost from one to three dollars; pebble glasses, six dollars.

When spectacles are first worn, they should not be employed steadily, only in the early morning or a dim light, or with fine print or sewing.

It is a very bad practice to keep the spectacles on all the time, in order to save trouble, for the eyesight fails much more rapidly under such circumstances, and those of greater power must be more speedily used. When the sight is beginning to fail, the eyes should be favored as much as possible; this can be done,

1st. By sitting in such a position as will allow the light to fall upon the page or sewing obliquely over the shoulder.

2d. By not using the eyes for such purposes by any artificial light, or before sunrise, or after sunset.

3d. By avoiding the special use of the eyes in the morning before breakfast.

4th. By resting them for half a minute or so, while reading or sewing, or looking at small objects, by looking at things at a distance or up to the sky, relief is immediately felt by so doing.

5th. Never pick any collected matter from the eye-lashes or corners of the eyes with the finger-nails; rather moisten it with the saliva and rub it away with the ball of the finger.

6th. Frequently pass the balls of the fingers over the closed eyelids, towards the nose; this carries off any excess of water into the nose itself by means of the little canal which leads into the nostril from each inner corner of the eye, which canal tends to close up in consequence of the slight inflammation which attends weakness of eyes.

7th. Keep the feet always dry and warm, so as to draw any excess of blood from the other end of the body.

8th. Use eye-glasses at first, carried in the vest-pocket, attached to a guard, for they are instantly adjusted to the eye with very little trouble; whereas, if common spectacles are used, such a process is required to get them ready, that to save trouble, the eyes are often strained to answer a purpose.

9th. Wash the eyes abundantly every morning. If cold water is used, let it be flapped against the closed eye with the fingers of the hand, not striking hard against the balls of the eyes. But it would seem a better plan to open the eyes in pure warm water, because warm water is more penetrating than cold; it dissolves much more readily and rapidly any hardened matter that may be about the lids, and is more soothing and more natural.

10th. The moment the eyes feel tired, the very moment you are conscious of an effort to read or sew, lay aside the book or needle, and take a walk for an hour, or employ yourself in some active exercise not requiring the close use of the eyes.

